# The Prevalence and Cost of Unapproved Uses of Top-Selling Orphan Drugs

**DOI:** 10.1371/journal.pone.0031894

**Published:** 2012-02-21

**Authors:** Aaron S. Kesselheim, Jessica A. Myers, Daniel H. Solomon, Wolfgang C. Winkelmayer, Raisa Levin, Jerry Avorn

**Affiliations:** 1 Division of Pharmacoepidemiology and Pharmacoeconomics, Department of Medicine, Brigham and Women's Hospital and Harvard Medical School, Boston, Massachusetts, United States of America; 2 Division of Rheumatology, Department of Medicine, Brigham and Women's Hospital and Harvard Medical School, Boston, Massachusetts, United States of America; 3 Division of Nephrology, Stanford University School of Medicine, Palo Alto, California, United States of America; University of Manitoba, Canada

## Abstract

**Introduction:**

The Orphan Drug Act encourages drug development for rare conditions. However, some orphan drugs become top sellers for unclear reasons. We sought to evaluate the extent and cost of approved and unapproved uses of orphan drugs with the highest unit sales.

**Methods:**

We assessed prescription patterns for four top-selling orphan drugs: lidocaine patch (Lidoderm) approved for post-herpetic neuralgia, modafinil (Provigil) approved for narcolepsy, cinacalcet (Sensipar) approved for hypercalcemia of parathyroid carcinoma, and imatinib (Gleevec) approved for chronic myelogenous leukemia and gastrointestinal stromal tumor. We pooled patient-specific diagnosis and prescription data from two large US state pharmaceutical benefit programs for the elderly. We analyzed the number of new and total patients using each drug and patterns of reimbursement for approved and unapproved uses. For lidocaine patch, we subcategorized approved prescriptions into two subtypes of unapproved uses: neuropathic pain, for which some evidence of efficacy exists, and non-neuropathic pain.

**Results:**

We found that prescriptions for lidocaine patch, modafinil, and cinacalcet associated with non-orphan diagnoses rose at substantially higher rates (average monthly increases in number of patients of 14.6, 1.45, and 1.58) than prescriptions associated with their orphan diagnoses (3.12, 0.24, and 0.03, respectively (p<0.001 for all)). By contrast, for imatinib, approved uses increased significantly over off-label (0.97 vs. 0.47 patients, p<0.001). Spending on off-label uses was highest for lidocaine patch and modafinil (>75%). Increases in lidocaine patch use for non-neuropathic pain far exceeded neuropathic pain (10.2 vs. 3.6 patients, p<0.001).

**Discussion:**

In our sample, three of four top-selling orphan drugs were used more commonly for non-orphan indications. These orphan drugs treated common clinical symptoms (pain and fatigue) or laboratory abnormalities. We should continue to monitor orphan drug use after approval to identify products that come to be widely used for non-FDA approved indications, particularly those without adequate evidence of efficacy.

## Introduction

The Orphan Drug Act encourages the development of medications to treat rare conditions. Manufacturers earn an orphan designation by demonstrating to the Office of Orphan Products Development (OOPD) at the Food and Drug Administration (FDA) that their product was directed at a disease affecting fewer than 200,000 people in the US [1]. The Act provides three primary incentives: 1) federal subsidies for clinical trials; 2) a tax credit of 50% of clinical research costs; and 3) an exclusive right to market the drug for seven years after approval. The market exclusivity period is highly valuable, since it begins at the time of approval, may run concurrently with—and even extend past—a drug's patent life [2], although unlike a patent, the market exclusivity is limited to the approved indication. Orphan designation also confers regulatory benefits, such as waiver of fees and expedited review by the FDA's Center for Drug Evaluation and Research (CDER) or the Center for Biologics Evaluation and Research (CBER). In the past 25 years, over 350 products designated as orphan drugs have subsequently been approved [3].

However, the Act has sparked some controversy [4]–[6]. Some researchers have pointed to “blockbuster” orphans that annually earn hundreds of millions, or billions, of dollars [7]–[8]. One previous study of orphan drug approval in treatment of HIV infection, for example, showed that manufacturers seek orphan drug designation for treatment of AIDS, although the wider community of HIV-infected patients used the drugs after approval, leading to substantial revenues for the manufacturer [9]. Some have questioned the appropriateness of providing additional publicly funded incentives to manufacturers of orphan products that become top sellers [10]–[11].

“Blockbuster” orphans arise in two main ways: through wide use of the orphan drugs outside their limited indications and through high per-unit costs. Though the FDA approves drugs for specific indications, physicians freely prescribe products for other purposes or patient populations (“off-label”) [12]. Off-label use has been reported in case studies of orphan drugs [13]. For example, epoetin alfa (Epogen) was originally approved as an orphan product in 1989 to treat anemia of end-stage renal disease, but was soon used widely in other forms of anemia [14].

High revenues for orphan products may also result from per-unit costs to patients or payers. The orphan drug imiglucerase (Cerezyme), an enzyme replacement for patients with Gaucher's disease (which affects about 1,500 US patients), can cost over $300,000 per patient per year [15]. High costs have been reported across numerous orphan drugs [16]–[17].

To examine whether top-selling orphan drugs are characterized by frequent off-label use or high costs (or both), we used a drug-disease dataset of medication use and clinical diagnoses in a large population of typical older patients. Our main hypothesis was that we would find statistically significant off-label, non-orphan use of these top-selling products.

## Methods

### Sources of data

We studied Medicare beneficiaries enrolled in 2 large state-funded programs of medication coverage for older patients: the Pharmaceutical Assistance Contract for the Elderly (PACE) program in Pennsylvania and the Pharmaceutical Assistance for the Aged and Disabled (PAAD) program in New Jersey from 1999 to 2005, when the introduction of Medicare Part D altered the availability of prescription use data through these state-based insurance programs. PACE and PAAD serve low-income adults 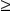
65 years of age, providing generous pharmaceutical benefits for virtually all prescription medications without restrictions, with a consistently small co-payment. We chose these databases because state programs, as well as the Medicare Part D program, are grappling with high medication costs for their elderly enrollees. During the study period, there were over 200,000 annual enrollees in each program. We linked these paid prescription claim records to Medicare Parts A and B claims data, which included information on recorded diagnoses. Studies have documented the positive predictive value of certain diagnoses (>94% for acute myocardial infarction) and accuracy of diagnosis dates (≥98% specificity for cancer diagnoses) with these databases [18]–[19].

### Study population

Participants were enrolled and active users of Medicare and either prescription drug benefit program for at least 6 months prior to their index date (defined below), as demonstrated by the program eligibility files. Patients must have filled at least 1 prescription and have had at least 1 health care encounter resulting in Medicare billing during this time period. The Institutional Review Board of Brigham and Women's Hospital approved this study; because it was a study of de-identified, already-collected data, the need to obtain informed consent was waived.

### Study drugs

The 100 top-selling pharmaceutical drugs by retail sales in 2009 included 12 approved for one or more orphan indications [20]. Among those products, 5 were targeted for orphan diseases at the time of first FDA approval. We excluded the remaining 7, for which the orphan indications were identified after the drug was already on the market, because prescribing trends for these drugs may be confounded by their market longevity before orphan approval. Thus, the drugs in our sample were: lidocaine patch (Lidoderm, Endo Pharmaceuticals, Chadds Ford PA), modafinil (Provigil, Cephalon, Frazer PA), cinacalcet (Sensipar, Amgen, Thousand Oaks CA), glatiramer (Copaxone, Teva, Petach Tikva, Israel) and imatinib (Gleevec, Novartis, Basel, Switzerland). We then excluded glatiramer because fewer than 50 patients who were prescribed this drug met our eligibility criteria during the study period. For the remaining four drugs, we used the FDA website to identify their dates of approval for orphan indications, as well as for any non-orphan indications [21]. The indications were linked to the corresponding International Classification of Diseases, Ninth Revision (ICD-9) diagnosis codes (Table 1). We obtained disease prevalence estimates from the OOPD via a Freedom of Information Act request. For cinacalcet, which is used in patients with chronic kidney disease and two laboratory abnormalities (hyperparathyroidism and hypercalcemia), we merged the main study database with the records from the US Renal Data System (USRDS), the national end-stage renal disease (ESRD) registry [22]. This merger permitted us to accurately distinguish patients with end-stage renal disease from patients with other chronic kidney disease when determining on- and off-label uses of this drug.

**Table 1:** Study drugs and FDA-approved indications (1998–2005).

Drug name (brand name)	FDA-Approved Indication	FDA Approval Date	Orphan Indication?	Estimated Orphan Disease Prevalence	ICD-9 code(s)
Lidocaine patch (Lidoderm)	Painful hypersensitivity and chronic pain in postherpetic neuralgia	March 1999*	Y	191,000	052, 053
Modafinil (Provigil)	Excessive daytime sleepiness in narcolepsy	December 1998	Y	120,000	347
	Shift-work sleep disorder	January 2004	N		327.36
	Adjunctive treatment of sleep apnea	January 2004	N		327.2, 780.57, 780.51, 780.53, 786.03
Cinacalcet (Sensipar)	Hypercalcemia in patients with parathyroid carcinoma	March 2004	Y	36,974	194.1, 237.4
	Secondary hyperparathyroidism in patients with chronic kidney disease on dialysis	March 2004	N		See USRDS
Imatinib (Gleevec)	Chronic myelogenous leukemia (CML)	May 2001	Y	42,000	205.1, 208.1, 758.89
	Gastrointestinal stromal tumor (GIST)	February 2002	Y	15,000	238.1, 238.9

*Lidocaine patch was not marketed by the manufacturer until September 1999.

USRDS = US Renal Data System (see reference 19).

### Identification and categorization of orphan drug prescriptions

The index date was the first use of each study drug by an enrollee in PAAD or PACE. After identifying filled prescriptions for each drug, we divided uses into three mutually-exclusive categories: “FDA-approved orphan use,” “FDA-approved non-orphan use,” and “non-FDA approved use.” FDA-approved orphan or non-orphan uses were defined as the presence of a diagnosis code for approved orphan or non-orphan indications up to 12 months before or 3 months after the index date. The expanded time period enabled conservative estimates of off-label use, given the rarity of orphan diagnosis codes. In the case of imatinib, approved for a second orphan indication in 2002 (gastrointestinal stromal tumor, GIST), we assigned uses associated with the supplemental orphan indication to the “FDA-approved orphan use” category after February 2002. Non-FDA approved uses were defined as all other uses. For each drug and category of use, we recorded the monthly numbers of patients (new and total) filling prescriptions and the total costs paid by the insurance programs.

The exposure period ran from market authorization of the drug to January 1, 2006, when Medicare Part D altered how enrollees received prescription benefits through these state-based programs. We contacted administrators at each program to characterize any unique restrictions on these products during the study period.

For each drug, we fit a linear regression model to estimate the monthly increase in the number of patients on each drug, by indication. The models for lidocaine patch included a spline term with knot at May 2004, when PACE restricted access to this drug. A spline term is a variable included as a predictor in a regression model to allow the slope on a given predictor (the increase in patients over time) to vary across regions of the predictor before and after the “knot” – that is, the time point of interest [23]. The models for modafinil included a spline term with knot at January 2004, when additional non-orphan indications were approved for this drug. In each model, the intercept was fixed at zero, since the number of filled prescriptions in month 0 (immediately before each drug went on the market) is known to be zero.

Using these models, we estimated the monthly increase in the number of patients on each drug by indication and time period. Confidence intervals were computed using bootstrap resampling, since the linear model assumption of independent, identically distributed errors is likely false. We also used bootstrap resampling to compute two-sided p-values to test the null hypothesis that the monthly increase in unapproved users equaled the monthly increase in approved orphan users in each time period. In addition, we calculated the average number of new users taking each drug by indication, where a new user was defined as a patient who had not filled a prescription for the index drug in the prior 180 days [24].

Finally, we subdivided unapproved uses of lidocaine patch, the most commonly prescribed drug in our sample, because some evidence-based guidelines recommend its use as first-line therapy for neuropathic pain apart from post-herpetic neuralgia [25]–[26], which is by definition an off-label use. We identified patients with diagnoses of neuropathic pain syndromes (excluding post-herpetic neuralgia) up to 12 months before their index date. Among the remaining patients, we noted those who received diagnoses of non-neuropathic pain syndromes up to 12 months before their index date. (See Appendix for a list of ICD-9 codes used to identify each subclass of non-FDA approved prescriptions.) We recorded the number of patients receiving at least one prescription and total costs paid during each month.

### National prescribing trends

The Centers for Medicare and Medicaid Services (CMS) provide aggregated quarterly drug spending data from each state Medicaid program [27]. We used this dataset to identify the total amount paid for the four study drugs by each state Medicaid program, including forty-nine states and the District of Columbia (Arizona data were not available) during the study period.

## Results

### Off-label use of orphan drugs

**Figure 1:**
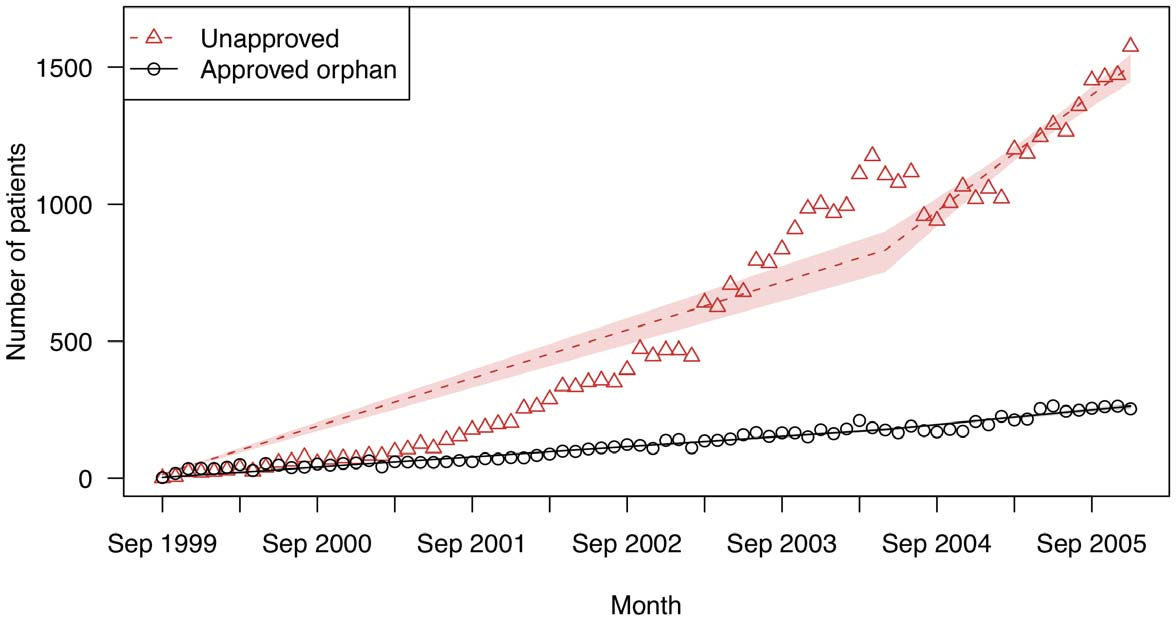
The number of patients filling prescriptions for lidocaine patch (Lidoderm) for on- and off-label uses in two state drug benefit programs. Raw data are plotted over the linear regression line. Shaded regions indicate bootstrapped 95% confidence intervals for the regression lines.

**Figure 2:**
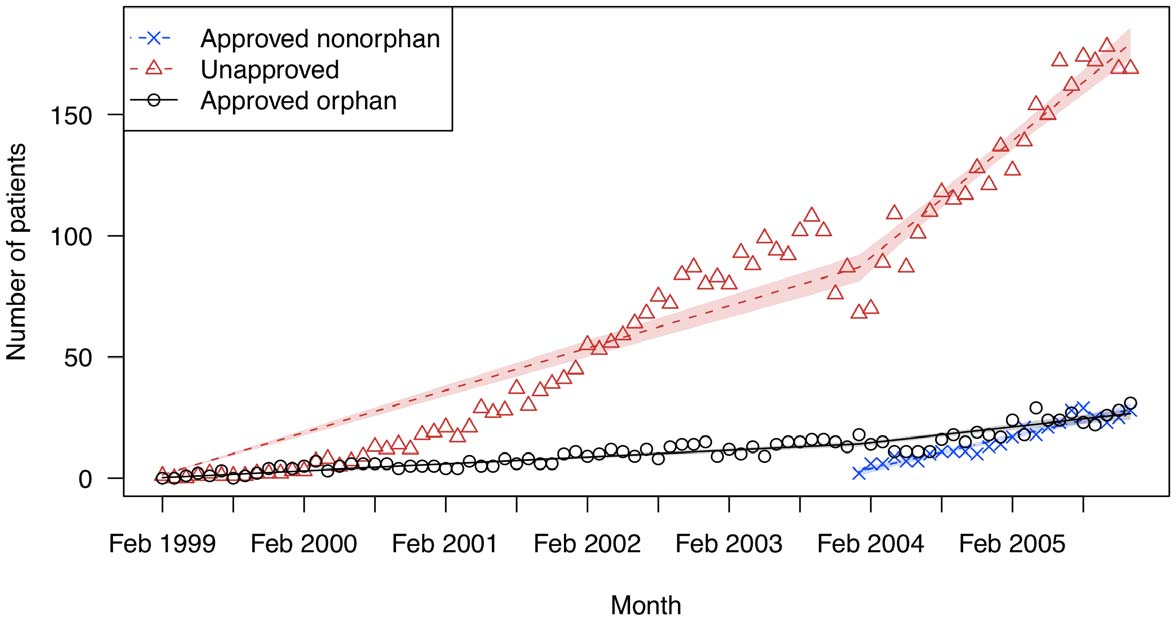
The number of patients filling prescriptions for modafinil (Provigil) for on- and off-label uses in two state drug benefit programs. Raw data are plotted over the linear regression line. Shaded regions indicate bootstrapped 95% confidence intervals for the regression lines.

**Figure 3:**
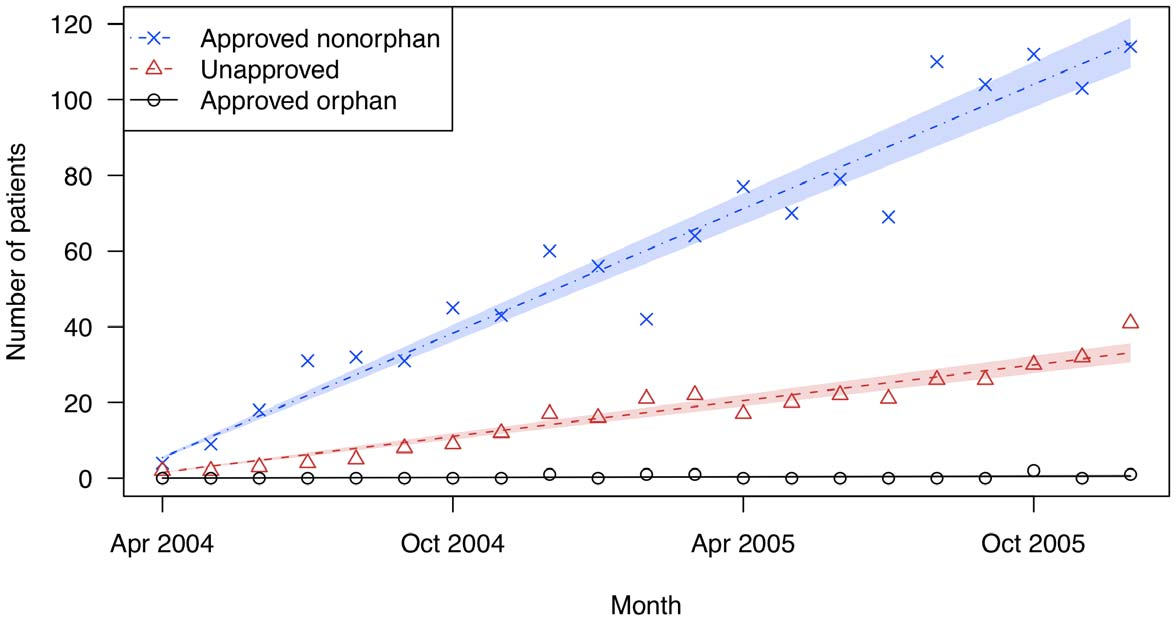
The number of patients filling prescriptions for cinacalcet (Sensipar) for on- and off-label uses in two state drug benefit programs. Raw data are plotted over the linear regression line. Shaded regions indicate bootstrapped 95% confidence intervals for the regression lines.

**Figure 4:**
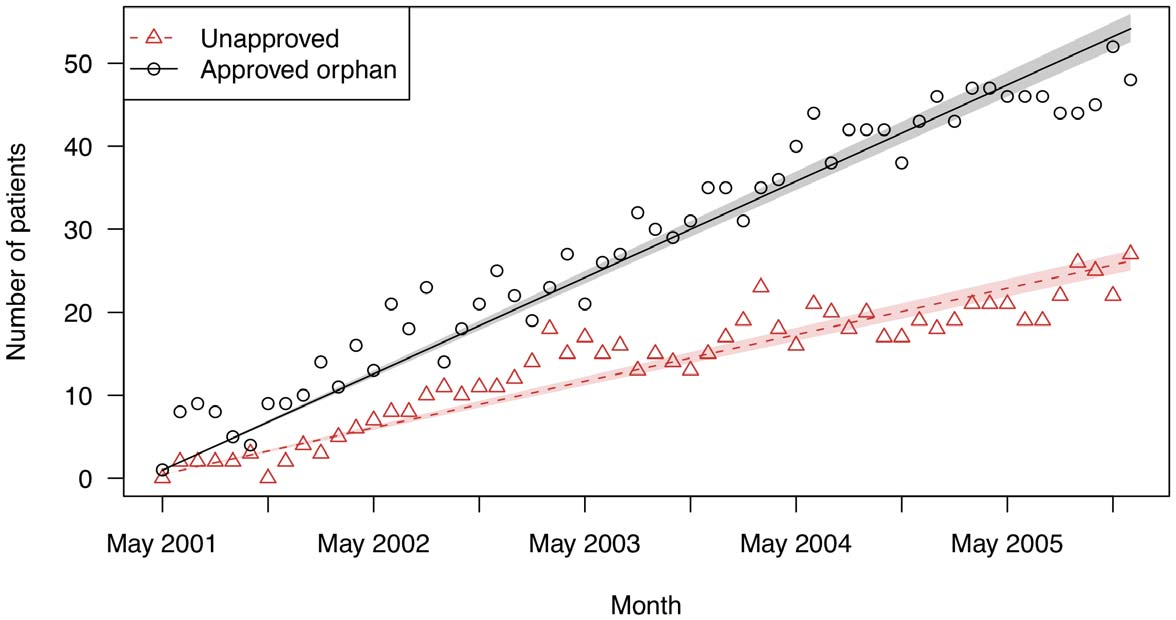
The number of patients filling prescriptions for imatinib (Gleevec) for on- and off-label uses in two state drug benefit programs. Raw data are plotted over the linear regression line. Shaded regions indicate bootstrapped 95% confidence intervals for the regression lines.

Figures 1, 2, 3, and 4 present the total number of patients filling prescriptions for each study drug by month and indication (trends in new patients tracked these results (data not shown)). For lidocaine patch, non-FDA approved uses far exceeded use by patients with post-herpetic neuralgia (82.3% vs. 17.7%; see Figure 1). The average monthly increase in use of lidocaine patch for off-label uses was 14.6 patients (95% confidence interval [CI]: 13.3–15.8), compared to only 3.12 (95% CI: 3.01–3.22) for the orphan use (p<0.001). In May 2004, the PACE program began to require confirmation of on-label use from the prescribing physician. Afterwards, off-label use in PACE dropped substantially; the subsequent rise (an average monthly increase of 35.3 patients (95% CI: 29.0–41.1)) reflects increasing use in PAAD patients only, which had no such restriction.

Modafinil showed a similar pattern (Figure 2). A minority (12.2%) of its use was for its on-label orphan indication (narcolepsy), while 87.8% of its use was in patients who did not have this diagnosis. The initial average monthly increase was 1.45 patients (95% CI: 1.35–1.54) for off-label uses, compared to only 0.24 (95% CI: 0.22–0.25) for the on-label use (p<0.001). Overall utilization increased in the beginning of 2004, when modafinil was approved for two supplemental non-orphan indications. However, the average monthly increase in patients did not change substantially for unapproved uses (2.56 (95% CI: 2.09–3.09)) or orphan uses (0.31 (95% CI: 0.18–0.41)).

Evaluation of cinacalcet was limited to 2 years of experience (Figure 3). For this drug, 1.2% of its use was for its approved orphan indication of hypercalcemia and parathyroid carcinoma, while 98.8% of use was for patients without these diagnoses (74.5% for the approved non-orphan indication and 24.3% for unapproved indications). The average monthly increase in patients for the FDA-approved orphan indication (hypercalcemia in patients with parathyroid carcinoma) was 0.03 (95% CI: 0.01–0.05). Use for the FDA-approved non-orphan indication (hyperparathyroidism in dialysis-dependent chronic kidney disease) remained the predominant use of the product during the study period (an average monthly increase of 5.48 patients (95% CI: 5.16–5.79) (p<0.001)), followed by unapproved uses (1.58 patients (95% CI: 1.46–1.70) (p<0.001)).

Finally, for imatinib (Figure 4), prescriptions for the orphan indications (61.3%) exceeded off-label use (38.7%). The average monthly increase was 0.47 patients (95% CI: 0.45–0.49) for its off-label uses vs. 0.97 patients (95% CI: 0.94–1.00) for its approved orphan uses (p<0.001).

### Costs of study drugs for public programs

Spending in PACE and PAAD for these products mirrored the trends identified among the prescription data. Overall, total spending for lidocaine patch was the greatest ($9.4 million), with $1.6 million for approved indications, and $7.8 million for unapproved indications. For modafinil, the two programs paid nearly $300,000 for approved uses, compared to more than $1 million for unapproved uses. For cinacalcet, approximately $50,000 was spent for approved orphan use, $400,000 for the approved non-orphan indication, and $130,000 for unapproved uses. By contrast, a total of $5.3 million was spent on imatinib, of which $3.6 million was for approved orphan uses and $1.7 million for other uses.

At a national level, state Medicaid programs spent considerable sums on the orphan drugs studied: $370 million for lidocaine patch (during 1999–2005), $156 million for modafinil (1999–2005), $74 million for cinacalcet (2004–2005), and $162 million for imatinib (2001–2005). If our calculations for relative on- and off-label expenditures are extrapolated to these national figures, we estimated $495 million in spending on off-label uses of these four orphan drugs by all state Medicaid programs combined.

### Uses of lidocaine patch for neuropathic pain

Off-label use of lidocaine patch was dominated by patients with diagnoses related to non-neuropathic pain. Initially, the observed monthly increase in patients using lidocaine patch was 10.2 patients with non-neuropathic pain diagnoses (95% CI: 9.2–11.1) and 3.6 patients with neuropathic pain diagnoses (95% CI: 3.2–3.9) (p<0.001). PACE and PAAD paid nearly $1.8 million for uses related to neuropathic pain, and $5.6 million for uses related to non-neuropathic pain (the remaining $0.4 million could not be classified in either category).

## Discussion

We found far greater use of two products – lidocaine patch and modafinil – for off-label indications than for any orphan indications. Use of cinacalcet was dominated by its FDA-approved non-orphan indication, but showed an increase in off-label use during the study period. By contrast, imatinib use was mostly related to its FDA-approved orphan indications. Thus, our hypothesis was only partially correct – statistically significant non-orphan and off-label use (compared to approved uses) was found in three of the four drugs we studied. Among all four drugs, we estimated that off-label use accounted for nearly $500 million in Medicaid expenditures, with spending increasing substantially during the exposure period.

The Orphan Drug Act has helped incentivize development of new drugs for rare conditions, although this study shows that there can be significant growth in off-label use of certain orphan drugs. The lidocaine patch and modafinil were approved to treat conditions manifested by common symptoms – respectively, post-herpetic neuralgia (pain) and narcolepsy (daytime sleepiness) – which can be intractable and frustrating conditions for both doctor and patient [28]–[29]. Although their pre-approval studies enrolled narrow populations covered by the Orphan Drug Act, these drugs showed efficacy in managing such symptoms in one context, so physicians may have been quick to prescribe them for other patients with similar symptoms. Similarly, cinacalcet was approved to treat laboratory abnormalities (hypercalcemia and hyperparathyroidism) found outside the populations for which it was originally approved. Unlike the other three drugs studied, imatinib was approved for distinct conditions (CML and GIST). Perhaps as a result, physicians were less likely to consider using these drugs off-label.

The growth of these drugs into top-sellers may be explained by other factors as well. Patients who present with chronic symptoms, such as pain or fatigue, may learn about newly approved products through media accounts and request prescriptions from their physicians. Some use of medications for non-FDA approved conditions has been illegally promoted by manufacturers; in the case of modafinil, for example, the manufacturer settled a lawsuit in 2008 for $425 million regarding alleged active promotion of its product for use outside of narcolepsy [30].

Growth of total sales of imatinib was not associated with off-label use, so could have been due to other factors, such as increases in the unit cost (approximately $56,000 per course/patient/year for GIST in 2009) [31]. Changes in unit costs also contributed to increases in spending on the study drugs. Despite the substantial commitment of resources through the Orphan Drug Act and other government funding to assist in the development of orphan drugs, their cost remains an important policy issue. For example, in 2009, 84% of Medicare Part D beneficiaries were enrolled in plans that put imatinib in a specialty tier with co-insurance rates as high as $1,366 per month [32]. Enacting limited waivers from state and federal antitrust laws could allow insurance plans to voluntarily band together in negotiating groups to seek lower drug prices for expensive orphan drugs where no alternative therapies exist [33].

Another way to reduce rates of unapproved non-evidence-based used of orphan drugs would be to vary the cost of the drug based on the indication. For example, payers could charge low co-pays to patients prescribed the drug to treat their orphan condition, and correspondingly high co-pays for non-evidence-based use. Applying such value-based insurance design to promote more appropriate use of orphan drugs, however, is limited by payers' ability to distinguish among the reasons that drugs are prescribed. As a result, orphan drugs are commonly priced the same for all indications. Some payers have tried to use administrative pre-certification forms for this purpose, although pharmaceutical companies selling orphan drugs have been investigated for allegedly teaching physicians how to fill out these forms to ensure approval of the drug for off-label uses [34].

This study has certain limitations. We studied top-selling drugs, so our conclusions do not generalize to all orphan-designated products. We also determined FDA-approved and non-approved uses from diagnosis codes submitted with billing claims from health care encounters. It is possible that some of the patients we identified as receiving a drug for unapproved indications may have had the indicated disease, but did not have a recorded diagnosis of it. Finally, the study population used to identify trends in utilization and spending comprised elderly persons with complete drug coverage residing in two states, and low-income patients in the Medicaid program. Our results may differ from other recipients of these agents.

In this analysis, we primarily focused on off-label use, rather than evidence-based use. However, for one of the products, the lidocaine patch, we found that an overwhelming share of the prescriptions were for patients with diagnoses for non-neuropathic pain syndromes, where no rigorous clinical trial evidence supports its use. Off-label use can have solid supporting evidence, and may be appropriate even in circumstances where gathering of supporting evidence can be difficult [35]. On the other hand, when non-FDA approved uses are not supported by adequate clinical evidence, patients do not receive the intended benefits from the drug, are less likely to be prescribed more effective treatments, and are exposed to risks of adverse events with no demonstrated countervailing benefits. The substantial costs of orphan products to Medicaid add to the concern about off-label and non-evidence-based uses. For the drugs in our sample, greater attention to implementing value-based insurance design may help limit non-evidence-based off-label use [36].

These findings documenting off-label use in certain top-selling orphan drugs may have important implications for the Orphan Drug Act and for drug development policy. Currently, generous orphan drug incentives in the US are earned during drug development or at the point of FDA approval, before the product reaches the market. The Orphan Drug Act has proven useful in encouraging the government and pharmaceutical manufacturers to devote resources towards developing new drugs for orphan conditions. Our data suggest that it might be preferable to continue to monitor orphan drug use after approval to identify products that come to be widely used for non-FDA approved indications – especially those for which there is also inadequate evidence of efficacy. Orphan drugs that quickly find widespread use are likely to provide an adequate return on investment to their manufacturers in a short time frame. When such use occurs widely, it may be reasonable to terminate the orphan drug market exclusivity period. The European version of the Orphan Drug Act contains a “clawback” provision that permits reduction of the statutory exclusivity period if the product is deemed sufficiently profitable, although it has never been invoked despite high prices and substantial revenues made by some orphan drug manufacturers in Europe [13]. Another alternative would be to seek reimbursement of the considerable government investments made in orphan drug development on the expectation that these drugs would find only limited use. The concept of reimbursing initial public investment in drug development remains controversial, although it has recently been endorsed by Francis Collins, Director of the National Institutes of Health [37].

Finally, our study suggests that regulators may be able to take a more proactive step and predict certain orphan products that are likely to be widely prescribed for non-FDA approved indications – those initially designed and approved to treat common symptoms or laboratory abnormalities, albeit in the context of a rare disease. For these products, it may be reasonable to withhold orphan drug status, and instead direct the limited resources of the program to encouraging development of novel products (such as imatinib) aimed at treating truly rare diseases. Applying incentives selectively to developing such products would adhere more closely to the original goals of the Orphan Drug Act.
